# Computational Molecular Modeling for Evaluating the Toxicity of Environmental Chemicals: Prioritizing Bioassay Requirements

**DOI:** 10.1289/ehp.11077

**Published:** 2008-02-01

**Authors:** James R. Rabinowitz, Michael-Rock Goldsmith, Stephen B. Little, Melissa A. Pasquinelli

**Affiliations:** National Center for Computational Toxicology, U.S. Environmental Protection Agency, Research Triangle Park, North Carolina, USA

**Keywords:** computational toxicology, docking, enrichment, false negatives, high-throughput screening, molecular modeling, prioritizing bioassays, virtual screening

## Abstract

**Background:**

The human health risk from exposure to environmental chemicals often must be evaluated when relevant elements of the preferred data are unavailable. Therefore, strategies are needed that can predict this information and prioritize the outstanding data requirements for the risk evaluation. Many modes of molecular toxicity require the chemical or one of its biotransformation products to interact with specific biologic macromolecules (i.e., proteins and DNA). Molecular modeling approaches may be adapted to study the interactions of environmental chemicals with biomolecular targets.

**Objective:**

In this commentary we provide an overview of the challenges that arise from applying molecular modeling tools developed and commonly used for pharmaceutical discovery to the problem of predicting the potential toxicities of environmental chemicals.

**Discussion:**

The use of molecular modeling tools to predict the unintended health and environmental consequences of environmental chemicals differs strategically from the use of the same tools in the pharmaceutical discovery process in terms of the goals and potential applications. It also requires consideration of the greater diversity of chemical space and binding affinity domains than is covered by pharmaceuticals.

**Conclusion:**

Molecular modeling methods offer one of several complementary approaches to evaluate the risk to human health and the environment as a result of exposure to environmental chemicals. These tools can streamline the hazard assessment process by simulating possible modes of action and providing virtual screening tools that can help prioritize bioassay requirements. Tailoring these strategies to the particular challenges presented by environmental chemical interactions make them even more effective.

A diverse spectrum of anthropogenic molecules is found in the environment, including chemicals introduced deliberately as well as unintended by-products of human activity. Through diligent monitoring, we are learning the identity, distribution, extent, and environmental persistence of these chemicals. To provide a reliable evaluation of the risk presented by these compounds, information about the specific molecules is required. This includes knowledge of the interaction of the chemicals with the environment and the effects of the chemicals or their successors on human health and ecologic systems.

The health and environmental effects of a chemical derive from a continuum of processes that proceed from the source of a chemical or its predecessors to a set of outcomes. However, it is often convenient to consider each process in the continuum as a discrete entity [U.S. Environmental Protection Agency (EPA) 2003]. Ideally, a risk assessment uses information relative to the specific chemical being considered. However, often the potential effects of a chemical must be evaluated when some relevant elements of the preferred data matrix are missing. In these situations, an estimate is derived by extrapolating from existing information.

Various approaches, including computational methods, have been developed to model these discrete steps in the source-to-outcome paradigm. These models provide approximations of the missing experimental information and a measure of the impact of specific missing data on the evaluation of risk. The models use existing information and can suggest new experiments. As a result, the source-to-outcome continuum becomes populated with information that includes experimental data, model-derived data, and connection models. The toxicant–target paradigm is a computational approach that employs molecular modeling methods to estimate relevant interactions and to populate the outcomes side of the source-to-outcome continuum.

## The Toxicant–Target Paradigm

The differential step in many mechanisms of toxicity may be generalized as the interaction between a small molecule (a toxicant) and one or more macromolecular targets. Targets include genetic material, receptors, transport molecules, and enzymes. In addition, other targets for toxicity could conceptually be described. The difference in activity observed between chemicals acting through the same biologic mode of action may then be understood as differences between their interactions with putative targets.

Some molecular modeling methods have been developed specifically to study interactions of this type and are commonly employed for the discovery of novel pharmaceutical agents (Coupez and Lewis 2006; Sousa et al. 2006). These methods can estimate the capacity of a chemical to interact with a specific target and cause a biologic effect. In the context of estimating chemical toxicity, this approach can yield predictions of the potential biologic activity. These molecular modeling tools can inform testing strategies or provide elements in a scheme for estimating toxicity that also include experimental results.

## Molecular Modeling in Computational Toxicology: Probing Toxicant–Target Structure

**Figure 1:**
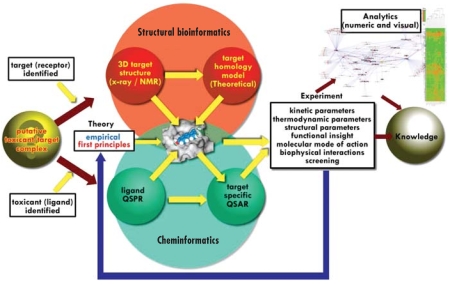
An overview of molecular modeling in computational toxicology. Abbreviations: QSAR, quantitative structure–activity relationship; QSPR, quantitative structure–property relationship. After the identification of a putative toxicant and target complexes (yellow sphere), the target structure (red spheres) is either experimentally determined or modeled based on structures with known sequence identity. Cheminformatics approaches and molecular docking (green spheres) can be used to obtain information about the putative toxicant (overlap of red and green spheres) and predict the desired properties, such as target-specific binding affinity and molecular modes of binding. Mathematical and visual analytics, such as hierarchical clustered heat maps or target-specific linkage maps, can yield knowledge that is chemical-class specific or target specific. Experimental guidance (blue arrow) optimizes this virtual screening approach.

The toxicant–target paradigm can be used to develop models for predicting chemical toxicity. These models are composed of approximate mathematical descriptions of the underlying physics and chemistry governing the behavior of the interacting molecules. These descriptions and their computational implementations construct a bridge between the information domains of experimental bio-molecular structure and biologic effects. Figure 1 depicts how molecular modeling can be used to estimate chemical toxicity via the toxicant–target paradigm.

Experimental information is used to provide a putative list of potential macromolecular targets related to chemical toxicity. For some of these targets, structural information is available or may be inferred from similar structures via homology modeling (Hillisch et al. 2004). The specific interactions between potential toxicants and the structures of known targets may be modeled via “docking” molecular modeling formalisms (Kuntz 1992).

In the absence of specific structural information about the targets, an alternative is to employ a ligand-based, cheminformatics strategy. This method derives relationships among various attributes of a database of ligands and known target-based activities. The attributes of the ligand may be simple or complex structural descriptions and properties that are either measured or derived computationally (Tong et al. 1997; Waller et al. 1996). Note that these cheminformatics methods have also been applied to predict chemical toxicity without direct consideration of a target (Prathipati et al. 2007), but methods of this type are not the primary subject of this report.

With both the structural bioinformatics and cheminformatics approaches, predictive models are developed and tested with experiments. A feedback process may be used to improve the quality of the predictions. In addition, these prediction tools can be used to identify important missing experimental information and relevant bioassays or properties that are currently unavailable. The underlying mechanism of action determines the range of applicability of the model. In order to use this approach as an element in a toxicity screen or for developing bioassay strategies, a number of choices must be made.

To a large extent, the pharmaceutical industry has driven recent advances in the design of molecular modeling tools for studying the interactions between a small molecule and a complex macromolecule (Jorgensen 2004). One approach for the discovery of leads for developing novel pharmaceutical agents employs computational “docking” of each member of a chemical library to macro-molecular targets that are chosen for potential therapeutic benefit. Molecular docking is designed to simulate the binding feasibility and affinity of small molecules to protein targets (Abagyan and Totrov 2001; Halperin et al. 2002). A docking calculation generates a variety of poses of a small molecule within a “binding region” of the macromolecular target, and typically includes ligand flexibility (Sousa et al. 2006). At times, some form of macromolecular flexibility (Carlson 2002) is also included. An important component of the docking simulation is to identify the potential binding sites within a macromolecular target. These sites could be an interior pocket or an indentation on the macromolecular surface (Huang and Schroeder 2006).

The calculation of a score assesses the potential relevance of each docking pose. Functions used for scoring poses typically take into account geometric shape complementarity as well as the physicochemical interactions between the small molecule and the macromolecular target (Coupez and Lewis 2006; Sousa et al. 2006). The docking score can be construed as a surrogate for the energy of interaction between the target and the small molecule, and in some cases is provided in terms of measures such as the log of the dissociation constant for inhibitor binding ( *K_i_*) or kilocalories per mole that may be directly compared with binding experiments. Comparison of these scores or computed interaction energies for a library of chemicals provides a means for ordering the molecules by their capacity to interact with the macro-molecular target. Chemicals with the best scores are most likely to interact with the target and are selected as subjects for further study. It is important to consider more than just a single pose with the best score because there are likely several local minimum energy poses in the interaction profile and a variety of highly ranked poses (Coupez and Lewis 2006; Sousa et al. 2006).

As is the case for the design of novel pharmaceutical agents, the successful application of docking methods to problems in chemical toxicity depends on the identification and availability of the crystal structures of the macromolecular targets or similar proteins. A variety of structures are available for macro-molecular targets that are known to be linked to the adverse effects of environmental chemicals, and their number is continually increasing (Hu et al. 2005; Wang et al. 2005). However, simulations of the interaction between small molecules and a macromolecular target for the purposes of drug discovery versus toxicity screening have distinct differences and, thus, present distinct challenges: *a*) the focus on different (yet overlapping) regions of chemical space; *b*) the strength of interaction between a small molecule and macromolecular targets; and *c*) the ultimate purpose of the virtual screening results.

**Figure 2:**
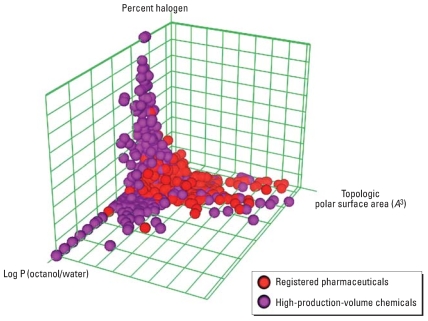
Plot of environmental anthropogenic compounds and registered pharmaceuticals subject to a Lipinski druglike filter. The axes represent three physicochemical characteristics for each compound: total polar surface area, partition coefficient (log P) between octanol and water, and fraction halogenated. The environmental compounds are the high-production-volume chemicals (Wolf et al. 2006), and the registered pharmaceuticals are the FDAMDD [FDA (Food and Drug Administration) maximum (recommended) daily dose] set from the DSSTox (Distributed Structure-Searchable Toxicity) database (Matthews et al. 2005).

Figure 2 is an approximate depiction of the chemical space for nonpharmaceutical commercial chemicals versus druglike chemicals in three selected dimensions of physicochemical characteristics. Viable drug candidates are typically those that have a strong interaction with a specific target, have good bioavailability, and are readily metabolized to inactive compounds and cleared from the system, in other words, compounds that have specific absorption, distribution, metabolism, excretion, and toxicity (ADMET) profiles and prescribed chemical properties. In contrast, environmental chemicals span a considerably larger chemical space and tread into “undesirable” property space from an ADMET perspective (too small, too insoluble, too reactive, etc.). They can also elicit adverse biologic effects from both strong and weak interactions with targets and in both a specific and nonspecific manner. Weak interactions and nonspecificity are also important aspects of pharmaceutical development because some side effects might arise from unintended binding to secondary targets (Ekins 2004; Ji et al. 2006). In addition, some environmental chemicals are produced and disposed of in significantly larger quantities than are pharmaceuticals and, hence, may present inadvertent human hazards over a long-term, low-dose exposure scenario. This is particularly the case if they are more chemically stable and persistent (i.e., resistant to metabolism), are potentially as bioavailable as drug candidates, or act through common pathways (thus posing cumulative effects) even if their individual target-specific interactions are much weaker than drugs or endogenous chemicals. Hence, evaluating the relative effectiveness of chemicals that bind more weakly or to multiple targets less specifically presents a greater challenge experimentally and computationally than does the discovery of novel pharmaceutical leads. Scoring functions in molecular modeling methods are typically optimized to identify chemicals that bind best to the target.

Another significant difference between pharmaceutical optimization and assessing the chemical toxicity of environmental chemicals is the purpose of an initial screen of a chemical library. For the pharmaceutical industry, the purpose of the initial screen for finding new drug candidates is to limit the number of chemicals that proceed to the next (more expensive) phase of testing while increasing the ratio of chemicals likely to become drugs to those likely to be inactive (i.e., increasing the “hit rate”). As long as the hit rate becomes significantly improved by this process, the exclusion of some active chemicals is a reasonable cost. In contrast, the purpose of an initial screen of environmental chemicals is to maximize the chance that active chemicals advance to the next phase of testing while eliminating as many inactive chemicals as possible. Given this objective and the corresponding uncertainties in assessing “potency” or activity based solely on computed scoring functions, the goal is to discover all or almost all of the agents having the potential to interact with the target, even those in significantly lower binding affinity domains than the endogenous or putative cognate ligand for the receptor. Thus, minimizing the number of false negatives is critical when screening environmental chemicals because the expectation is that positive chemicals will be tested later in an experimental protocol. A toxicity screen should not reject a compound (i.e., classify as inactive or safe) that has a weak affinity for a target or multiple targets without considering its ADMET properties, persistence, and chance of exposure. Obtaining activity signatures from receptor affinity profiles of compounds not intended for therapeutic application may become an important aspect of multilevel screening programs that include measured biologic properties, such as ToxCast (Dix et al. 2007).

## Enrichment and False Negatives

**Figure 3:**
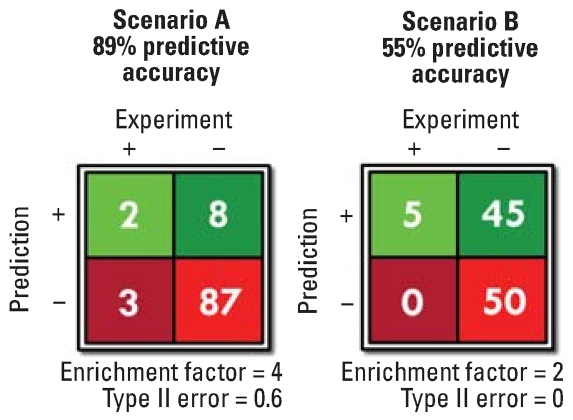
Illustration of type II errors and enrichment factors in chemical screening. The statistical “type II error” is the ratio of the number of false negatives to the sum of false negatives and true positives. The “enrichment factor” is the ratio of the true positive rate of the screen (the number of true positives divided by the number of true positives plus false positives) to the ideal positive rate of the chemical library (the number of positive chemicals in the library divided by the number of chemicals in the library).

Figure 3 shows two hypothetical data scenarios derived from computational docking experiments using the same library of chemicals against a model target. The difference between the two sets arises either from choosing different docking score thresholds between predicted active and inactive chemicals or from using different scoring functions. For this example, definitive (ideal) experimental tests determine that 5% of the chemicals are active and 95% are inactive relative to the macromolecular target of interest. Scenario A has 89% of the chemicals classified correctly, whereas scenario B has only 55% of the chemicals classified correctly. The enrichment factor for scenario A is 4 because 20% of the chemicals selected for further testing (i.e., screened positive) will prove to be positive, whereas the enrichment factor for scenario B is only 2. However, the type II error for scenario A is 0.6, whereas it is 0.0 for scenario B.

The screening method used for scenario A appears to be better by many measures and is an appropriate approach if the goal is to discover novel pharmaceutical leads. On the other hand, the screening method used for scenario B is more appropriate when screening chemicals for potential toxicity. Scenario B will carry many more chemicals to the next phase of testing, but the negatives are true negatives. Chemicals identified by this pre-screen as negative will have a lower priority for continued testing and perhaps will not be tested in any other manner for effects at this particular target.

This discussion addresses the challenges in using current docking methods for assessing chemical toxicity. The methods that are currently available for computational molecular docking were developed for drug discovery and therefore are optimized to screen large chemical databases to find the most active molecules and increase the enrichment factor. Some false negatives are not an important concern as long as the enrichment rate is significantly increased. In contrast, a screen for assessing chemicals for potential toxicity often deals with a smaller database of chemicals (the chemicals encountered in the environment) and must be capable of identifying chemicals with much lower affinities than the natural ligands. Therefore, scoring functions and/or methods for delineating active chemicals from inactive chemicals must be explored and better understood in the context of environmental chemicals, and may involve computational methods that are more accurate but computationally intensive.

## Virtual Screening of Chemicals

The usual approach for virtual screening of chemicals for toxicity is to screen a database of chemicals for each chemical’s capacity to interact with a single macromolecular target and initiate a single mode of chemical toxicity. A virtual screen that is receptor specific produces a score vector where each element represents the interaction of that receptor with a different chemical entity. Inverting the problem so that the vector now contains elements that represent the capacity of a single chemical to interact with each of a series of targets allows the most likely targets and, therefore, the most likely modes of toxicity for a specific chemical to be identified. A matrix is produced by interrogating a library of targets with a database of chemicals. The relationships among the elements in this matrix have the potential to yield additional insights, such as receptor cross-talk or multiple modes of biologic potency (Macchiarulo et al. 2004), and modes of sequestration (Perry et al. 2004). A combination of these computationally derived data and experimentally derived data can be data-mined to extract patterns and associations. These associations can provide additional knowledge for assessing the hazards of chemicals and chemical mixtures or be used to improve prediction tools in the context of toxicity such as scoring functions for molecular docking calculations.

For some targets in the library, other interactions in addition to those included in docking algorithms must be considered. For instance, modes of toxicity have been identified that require covalent interactions between the toxicant and the target (Zhou et al. 2005) or that necessitate the redistribution of charge in both the toxicant and the target (Pardo et al. 1993). These interactions involve the electronic structure of both the putative toxicant and target molecules and, hence, require some level of quantum chemistry. However, most current docking methods include only classical interactions. One approach is to use molecular docking to determine the structure of complexes, and then to calculate the short-range interactions with quantum chemistry methods. A few attempts have been made in recent years to build essential quantum effects directly into molecular docking calculations, such as quantum polarized ligand docking (Cho et al. 2005).

In addition, to take into account the known or hypothesized biotransformation products during the molecular docking calculations, each constituent must be included as separate chemical entities in the docking calculations. Computational tools already exist for predicting metabolites (Jolivette and Ekins 2007), so docking calculations could be improved by networking with metabolism prediction models.

Another application for virtual screening is predicting prospective targets for a particular chemical and its metabolites using inverse docking strategies. In drug discovery, this approach can identify potential alternate uses for drug candidates or predict side effects of pharmaceuticals that might arise from unintended interactions with other targets (i.e., off-target effects), thus producing adverse outcomes. As an element in a toxicity screen for environmental chemicals, inverse docking tools can be used to guide experimental testing. Inverse docking can help focus efforts and lead to a reduction in the use of resources as well as the time required for a hazard or risk assessment. Some attempts at inverse docking methods have arisen in recent years (Ekins 2004; Ji et al. 2006), although these methods still face some limitations that prevent their more general use in virtual screenings. Inverse docking strategies could become a more viable resource as further target crystal structures become available and molecular docking methods are improved, and in conjunction with systems biology methods such as proteomics and genomics (Loging et al. 2007).

## Conclusions

Computational molecular modeling methods aid the risk assessment process by providing a rational approach for some extrapolations in the evaluation of chemical hazard. For instance, when elements of a data set required for evaluating the potential hazard of a chemical are unavailable and inferences can be made based on interactions with putative targets, molecular modeling can be used to simulate the relevant missing information. Both ligand-and structure-based molecular modeling methods used in pharmaceutical discovery can be adapted to provide this type of simulated data. However, because of the greater diversity of chemical space and binding affinity domains being considered and the differences in the strategic application of the results (the need to minimize false negatives), these molecular modeling strategies require additional considerations when assessing chemical hazards. Molecular docking of potential environmental chemicals to putative macromolecular targets for toxicity provides a measure of their capacity to interact and hence is an aid in the (pre)screening process for specific modes of toxicity. These results provide a rationale for developing further, more complete testing strategies.
